# 2-Methyl-5H-benzo[d]pyrazolo[5,1-b][1,3]oxazin-5-imine, an edaravone analog, exerts neuroprotective effects against acute ischemic injury *via* inhibiting oxidative stress

**DOI:** 10.7555/JBR.32.20180014

**Published:** 2018-04-20

**Authors:** Huanyu Ni, Yixuan Song, Haiyin Wu, Lei Chang, Chunxia Luo, Dongya Zhu

**Affiliations:** 1. Institution of Stem Cells and Neuroregeneration;; 2. Department of Pharmacology, School of Pharmacy;; 3. The Key Laboratory of Precision Medicine of Cardiovascular Disease, Nanjing Medical University, Nanjing, Jiangsu 211166, China.

**Keywords:** neuroprotection, oxidative stress, scavenging activity, sensorimotor function, spatial memory, stroke

## Abstract

Oxidative stress plays an indispensable role in the pathogenesis of cerebral ischemia. Inhibiting oxidative stress has been considered as an effective approach for stroke treatment. Edaravone, a free radical scavenger, has been shown to prevent cerebral ischemic injury. However, the clinical efficacy of edaravone is limited because it has a low scavenging activity for superoxide anions (O_**2**_^·−^). Here, we report that 2-methyl-5H-benzo[d]pyrazolo[5,1-b][1,3]oxazin-5-imine, a novel small-molecule compound structurally related to edaravone, showed a stronger inhibitory effect on oxidative stress *in vitro*. *In vivo*, 2-methyl-5H-benzo[d]pyrazolo[5,1-b][1,3]oxazin-5-imine reversed transient middle cerebral artery occlusion-induced dysfunctions of superoxide dismutases and malondialdehyde, two proteins crucial for oxidative stress, suggesting a strengthened antioxidant system. Moreover, 2-methyl-5H-benzo[d]pyrazolo[5,1-b][1,3]oxazin-5-imine decreased blood brain barrier permeability. Then, we found that 2-methyl-5H-benzo[d]pyrazolo[5,1-b][1,3]oxazin-5-imine had a stronger neuroprotective effect than edaravone. More importantly, 2-methyl-5H-benzo[d]pyrazolo[5,1-b][1,3]oxazin-5-imine decreased not only infarct size and neurological deficits in the acute phase but also modified neurological severity score and escape latency in Morris water maze task in the delayed period, indicating enhanced neuroprotection, sensorimotor function and spatial memory. Together, these findings suggest that 2-methyl-5H-benzo[d]pyrazolo[5,1-b][1,3]oxazin-5-imine could be a preferable option for stroke treatment.

## Introduction

Ischemic stroke remains a vexing public health problem and it is estimated that the costs of stroke care will account for 6.2% of the total burden of illness in 2020^[1]^. Currently, recanalization and neuroprotection are the two main strategies for ischemic stroke treatment. Tissue plasminogen activator (tPA), which can promote thrombolysis by activating the endogenous fibrinolytic system, is the only FDA approved agent for acute ischemic stroke (AIS)^[2]^. Unfortunately, the narrow time window and the risk of symptomatic intracerebral hemorrhage limit the clinical use of tPA. In clinical trials, few encouraging preclinical results of neuroprotectants have been translated into positive outcomes^[3]^. Therefore, it heightens the need to develop new pharmacological treatments for stroke patients.

Oxidative stress is a major component of the ischemic stroke cascade^[4]^. Free radicals are endowed with highly reactive activity, causing the oxidation of other molecules, including DNA, lipids and proteins^[5]^, and thus leading to ischemic injury^[6^‒^7]^. This process indicates that free radicals could be a valid target for stroke treatment^[8]^. Reactive oxygen species (ROS), including superoxide anions (O_**2**_^·−^), hydroxyl radicals (HO**·**), and hydrogen peroxide (H_2_O_2_), are generated during normal cellular respiration and metabolic processes^[9]^, physiologically serving as signaling molecules. Brain appears particularly vulnerable to free radical-mediated attacks, due to its limited antioxidant defenses^[10]^ and heavy demand for oxygen. Acute ischemia leads to the activation of ROS-generating enzymatic systems, including NADPH oxidases (NOXs), respiration chain in the mitochondria and xanthine oxidase^[11^‒^13]^, thereby causing noxious effects^[14]^. Recently, it has been well accepted that NOXs generate the majority of ROS, especially O_**2**_^·−^ in AIS^[15]^. Notably, in our previous work, inhibiting NOXs was confirmed to attenuate oxygen and glucose deprivation-induced damage *in vitro*^[16]^, making NOXs the target for neuroprotectants.

Phenolic compounds with keto-enol tautomerism were identified with antioxidant property. Among them, edaravone showed a strong scavenging activity for ROS. Since then, an increasing number of pre-clinical studies have reported that edaravone significantly attenuated acute ischemic damage *via* scavenging ROS and inhibiting ROS-induced deleterious signaling cascade^[6^‒^7^,^17^‒^19]^, including lipids peroxidation, blood brain barrier disruption, and neuronal and vascular damage. Though antioxidant activity is a major effect of edaravone against acute ischemic injury^[20]^, other mechanisms, such as the counteraction of microglia-induced inflammation and neurotoxicity^[21^‒^22]^, may also account for the edaravone-induced improvements in stroke outcomes, to some extent. In 2001, edaravone was approved in Japan as a neuroprotectant for treatment of AIS^[23]^. There has been wide agreement that O_**2**_^·−^ can react with nitric oxide (NO) to produce peroxynitrite (ONOO^−^), a stronger oxidative radical, and lead to protein nitration and dysfunction^[9]^. However, edaravone was reported to have no major scavenging effect on O_**2**_^·−[17^,^19]^, thus probably limiting its potency. Here, we used edaravone as a leading compound, designed and synthesized a novel compound (2-methyl-5H-benzo[d]pyrazolo[5,1-b][1,3]oxazin-5-imine), and named it TR. We thus wonder whether TR exerts stronger neuroprotective effects against acute ischemic injury *via* inhibiting oxidative stress.

## Materials and methods

### Animals

Adult male Sprague-Dawley rats (250‒300 g; B&K Universal Group Limited, Shanghai) were used. Rats were raised in an air-conditioned room [(20±2) °C, 12 hours light/dark circle] with food and water* ad libitum*. The experimental protocol was approved by the Institutional Animal Care and Use Committee of Nanjing Medical University.

### Reagents and drugs

5,5-Dimethyl-1-pyrroline N-oxide (DMPO), hypoxanthine, xanthine oxidase and 2,3,5-tripenyltetrazolium chloride (TTC) were purchased from Sigma-Aldrich (St. Louis, USA). Edaravone and 2-methyl-5H-benzo[d]pyrazolo[5,1-b][1,3]oxazin-5-imine were supplied by Simcere Pharmaceutical Group and Nanjing Zhongrui Pharmaceutical Co., Ltd., respectively. Assay kits for superoxide dismutases (SODs) and malondialdehyde (MDA) were obtained from Jiancheng Bioengineering Institute (Nanjing, China).

### Synthesis of 2-methyl-5H-benzo[d]pyrazolo[5,1-b][1,3]oxazin-5-imine

For synthesis of 2-methyl-5H-benzo[d]pyrazolo[5,1-b][1,3]oxazin-5-imine, 2-aminobenzonitrile (59 g, 0.9 mol) was mixed with concentrated hydrochloric acid (600 mL) and crushed ice (500 mL), and the mixture was vortexed until completely dissolved. Under −7 °C to −3 °C, the solution was added dropwise with a solution of sodium nitrite (34.5 g) in H_2_O (150 mL), and stirred for 10 minutes to produce an orange-yellow liquid. Stannous chloride (350 g) and concentrated hydrochloric acid (1,000 mL) were mixed and stirred until completely dissolved. Under −5°C, the solution was added dropwise with the above product to present the white sediment, and stirred for 2 hours. Precipitated solid was isolated by filtering the reaction mixture and dried to obtain 2-hydrazinylbenzonitrile hydrochloride.

The intermediate 2-hydrazinylbenzonitrile hydrochloride (8.5 g) and ethyl acetoacetate (6 g) were dissolved in methanol (100 mL), and the solution was added with a solution of 50% sodium methanolate (5.5 g) in CH_3_OH (50 mL) and stirred for 10 minutes. The mixture was heated to reflux for 6 hours, and then filtered to obtain the solid, which was eluted with methanol (20 mL). The eluent was added with H_2_O (200 mL) to precipitate solid, and then filtered. The residues were washed with water to obtain the buff solid (7 g).

The intermediate 1-(2-Cyanophenyl)-3-methylpyrazole-5-one (20 g) was dissolved in anhydrous tetrahydrofuran (200 mL). The dry hydrogen chloride gas was passed into the solution until saturation. The mixture was stirred at room temperature overnight, and then concentrated under reduced pressure. The product was added with anhydrous tetrahydrofuran (100 mL) and anhydrous sodium acetate (10 g). Then, the mixture was stirred at room temperature for 1 hour. The filtrate was obtained by filtering the reaction mixture, concentrated under reduced pressure, and recrystallized with ethyl acetate (13 g).

### Electron spin resonance (ESR)

TR and edaravone quenching activities for O_**2**_^·−^ and HO**·** were determined by an ESR spectrometric method^[19]^. Measurements were performed using a Bruker EMX-10/12 ESR spectrometer operating at X-band with a TE 102 cavity with a capillary. The magnetic field, sweep width, microwave frequency, power, modulation frequency, modulation amplitude, temperature, gain and sweep time was 347 mT, 20 mT, X-band, 20 mW, 100 kHz, 0.1 mT, 25 °C, 5 × 10^4^, and 84 seconds, respectively.

For the detection of O_**2**_^·−^, 20μL of 0.7 U/mL xanthine oxidase, 40μL of 4.4 mmol/L hypoxanthine, 40μL of 1.0 mmol/L DTPA, 60μL of 250 mmol/L DMPO, 20μL of PBS, and 20μL of TR or edaravone (24 mmol/L, 12 mmol/L, 6 mmol/L, 3 mmol/L, 1.5 mmol/L) were mixed at room temperature. The amount of DMPO-OOH spin adduct formed was measured within 2 minutes.

For the detection of HO**·**, 50μL of 4 mmol/L FeSO_4_ solution, 50μL of TR or edaravone (12 mmol/L, 6 mmol/L, 3 mmol/L, 1.5 mmol/L, 0.75 mmol/L, 0.375 mmol/L), 50μL of 250 mmol/L DMPO and 50μL of 40 mmol/L H_2_O_2_ were mixed in a test tube. The amount of DMPO-OH spin addition formed was estimated exactly at 30 seconds after adding H_2_O_2_. The signal intensity was evaluated by the relative peak of the second special signal of the quartet of the DMPO-OH spin adduct.

### Surgical preparation

Transient middle cerebral artery occlusion (tMCAO) and permanent middle cerebral artery occlusion (pMCAO) were performed in rats as described previously^[19]^. Briefly, under ketamine anesthesia, a 4/0 surgical nylon monofilament with rounded tip was introduced into the left internal carotid artery through the external carotid stump, advanced 20‒21 mm past the carotid bifurcation until a slight resistance was felt. The filament was left in place for 120 minutes and then withdrawn for reperfusion. Sham-operated rats received the same procedure as tMCAO rats except that the occluding filament was inserted only 7 mm above the carotid bifurcation. For pMCAO preparation, the operation was the same as tMCAO except that the filament was not withdrawn.

### SODs and MDA measurement

Rats were subjected to tMCAO for 120 minutes, treated with TR, edaravone or vehicle (6 mg/kg, i.v.) immediately after reperfusion and decapitated at 24 hours after reperfusion. The ipsilateral hemispheres were dissected and homogenized in 6 mL physiological saline. The homogenates were centrifuged at 3,500 r/minute for 15 minutes at 4 °C. The concentrations of SOD and MDA were measured in the supernatant, using commercially available kits (Jiancheng Bioengineering Institute, Nanjing, China) and expressed as U/mg protein and mmol/mg protein, respectively.

### Blood brain barrier (BBB) permeability

To test the BBB permeability, we treated tMCAO rats with TR, edaravone or vehicle (6 mg/kg, i.v.) immediately after reperfusion. Evans blue (4%, 3 mL/kg) was infused intravenously at 22 hours after reperfusion. Two hours later, the rats were completely perfused with saline. After decapitation, the brains were removed rapidly and divided into contralateral and ipsilateral hemispheres. Each hemisphere was weighed, homogenized in 2 mL of 50% trichloroacetic acid, and centrifuged at 10,000 r/minute for 20 minutes. The extracted dye was diluted with ethanol (1:3), and its fluorescence was determined with a luminescence spectrophotometer (610 nm). Evans blue contained in tissue was quantified from a linear standard line and was expressed as Evans blue (µg) / tissue (g).

### Infarct size measurement, neuroscore assessment, modified neurological severity score (mNSS) test

The infarct volume measurement, neuroscore assessment and mNSS test were performed as described^[24^‒^26]^. Infarct volume was expressed as a percentage area of the coronal section in the infarcted hemisphere. In brief, brains were removed rapidly and frozen at −20 °C for 5 minutes. Coronal slices were made at 1‒2 mm from the frontal tips, and sections were immersed in 2% 2,3,5-tripenyltetrazolium chloride (TTC; Sigma T8877) at 37 °C for 20 minutes. Neuroscore assessment was performed based on a five-point scale system. (Rating scale: 0= no deficit, 1= failure to extent left forepaw, 2= decreased grip strength of left forepaw, 3= circling to left by pulling the tail, and 4= spontaneous circling).

To evaluate the long-term effects of TR on sensorimotor function, mNSS test was performed^[26]^. In the severity scores of impairment, one point was scored for inability to perform the task or lack of proper response for a given reflex; thus, a higher score meant a more severe injury. The score was graded from 0 to 18 (normal score, 0; maximal deficit score, 18). Severe injuries were indicated by a score range of 13 to 18, moderate injuries 7 to 12, and mild injuries 1 to 6.

### Morris water maze task (MWM)

The spatial cognitive performance of rats was evaluated by MWM, as described in our previous reports^[19]^. A circular swimming pool (Jiliang Neuroscience Inc., Shanghai, China) measuring 180 cm in diameter and 48 cm in height was filled with water to a depth of 26 cm at (24±2) °C. Four starting points around the edge of the pool were designated as N, E, S, and W, dividing the pool into four quadrants. A platform, 10 cm in diameter, was located in a constant position in the middle of one quadrant. To render it invisible to the rats, the platform was submerged 2.0 cm below the surface of the water. The task for the rats was to escape from the water by locating the hidden platform. Two days before training, the animals were habituated to swimming for 60 seconds in the pool without a platform. One block of four trials was given for 4 consecutive days. For each trial, the rats were placed in the water facing the wall of the pool at one of the four starting points and allowed to swim for a maximum of 90 seconds. If the rats found the platform, they were allowed to stay on it for 10 seconds; the rats who failed to find the platform were guided to it and allowed to stay there for 10 seconds. Each trial was videotaped *via* a ceiling-mounted video camera and the animal's movement was tracked using Ethovision software (Noldus Information Technology, Wageningen, The Netherlands), which allows the measurements such as latency (time to reach the platform) and swimming speed. On the next day, the rats were given one 60-second retention probe test with the platform removed from the pool. During retention, the time spent in the target quadrant was measured.

### Statistical analysis

Data were presented as mean±SEM. Statistical analysis was performed using SPSS software (version 22). Comparisons among multiple groups were made with one-way or two-way analysis of variance (ANOVA) , followed by Scheffe post hoc test. Behavioral data collected at repeating time points were analyzed by two-way repeated measures ANOVA, followed by Bonferroni post hoc test. *P*<0.05 was considered statistically significant.

## Results

### TR effectively scavenges free radicals *in vitro*

**Fig.1:**
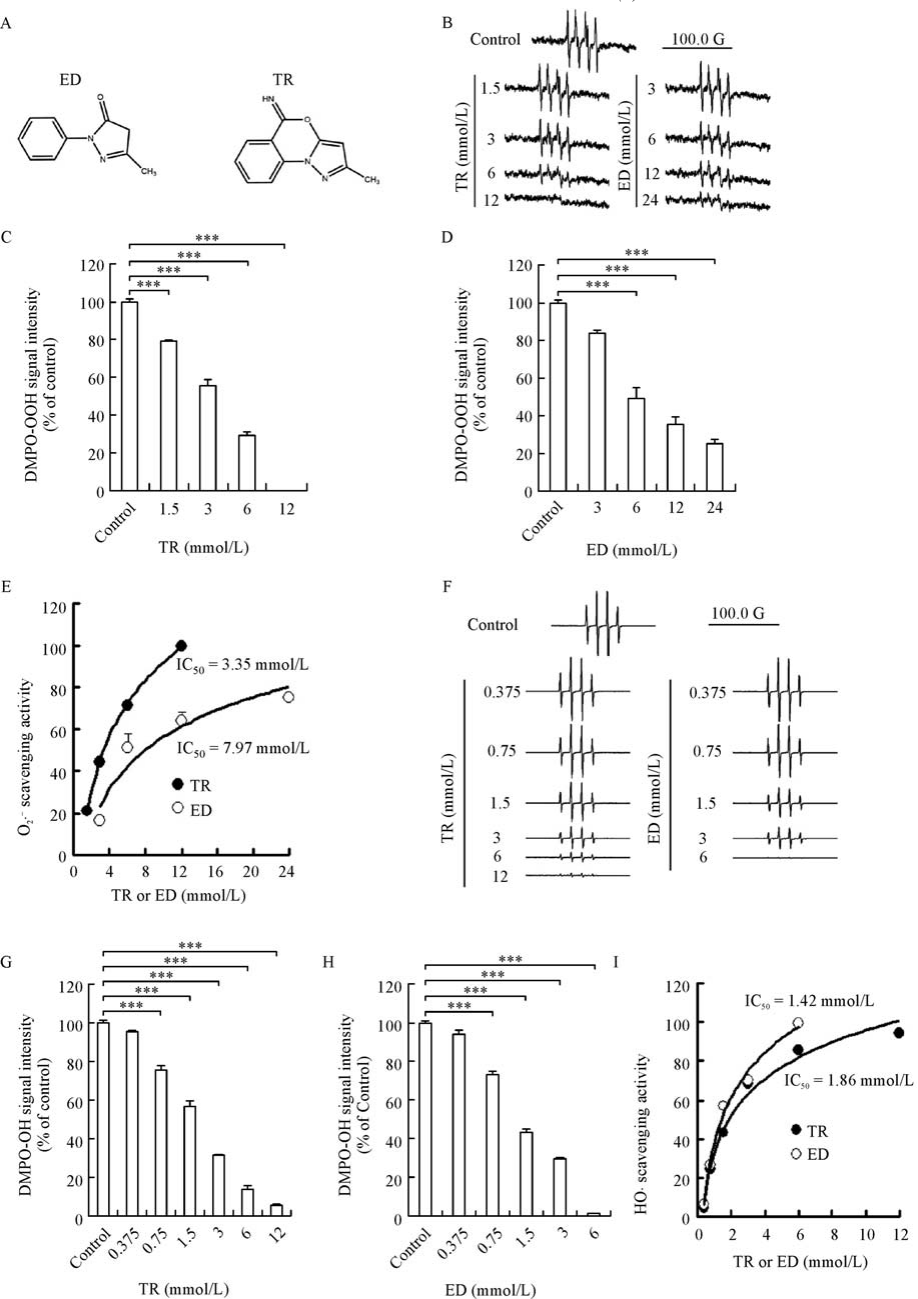
**Scavenging effects of 2-methyl-5H-benzo[d]pyrazolo[5,1-b][1,3]oxazin-5-imine (TR) on ROS ***in vitro***.** A: Structures of edaravone (ED) and TR. B–E: O_**2**_^·−^ scavenging activity of TR and ED (n = 3 per group). B: Representative ESR spectra of DMPO-OOH signal showing scavenging effects of TR and ED on O_**2**_^·−^ at the indicated concentrations. C–D: Bar graphs showing DMPO-OOH signal intensity. E: Fitted concentration-response curve showing the O_**2**_^·−^ scavenging activity. F–I: HO· scavenging activity of TR and ED (n = 3 per group). F: Representative ESR spectra of DMPO-OH signal showing scavenging effects of TR and ED on HO· at the indicated concentrations. G–H: Bar graphs showing DMPO-OH signal intensity. I: Fitted concentration-response curve showing the HO· scavenging activity. ED: edaravone; ESR: electron spin resonance; ROS: reactive oxygen species. ***P < 0.001 vs. Control.

Edaravone has been shown as a neuroprotectant in clinical practice^[20]^, due to its scavenging activity of ROS. TR was designed and synthesized to be structurally related to edaravone (***Fig. 1A***). So, we hypothesize that TR has the ability to scavenge ROS, as edaravone does. To test this, we detected signal intensities of DMPO-OOH adduct and DMPO-OH adduct, indicators of O_**2**_^·−^ and HO**·**, respectively, using an ESR spectrometric method. As expected, TR and edaravone effectively scavenged O_**2**_^·−^ dose-dependently (***Fig. 1B‒D***). Meanwhile, we compared the scavenging effects of the two drugs on O_**2**_^·−^, and calculated that half maximal inhibitory concentration (IC_50_) of TR and edaravone was 3.35 and 7.97 mmol/L (***Fig. 1E***), respectively, suggesting that the scavenging effect of TR on O_**2**_^·−^ was 2.4-fold stronger than that of edaravone. Moreover, we also found that TR and edaravone scavenged HO**·** in a dose-dependent manner (***Fig. 1F-H***) with comparable ability, as indicated by their IC_50_ of 1.86 and 1.42 mmol/L (***Fig. 1I***), respectively.

### TR significantly prevents tMCAO-induced focal cerebral ischemia

**Fig.2:**
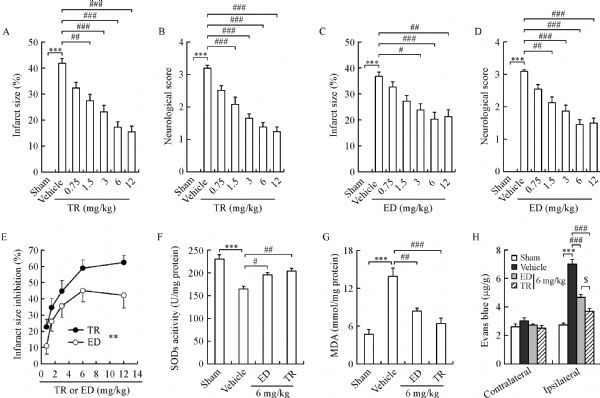
**TR significantly prevents acute cerebral ischemia.** Rats were subjected to tMCAO for 120 minutes, treated with TR, edaravone (ED) or vehicle (i.v.) immediately after reperfusion. We tested neurological score, infarct size, SOD activity, MDA concentration and the integrity of blood brain barrier (BBB) at 24 hours after reperfusion. For BBB experiment (H), Evans blue was given (4%, 3 mL/kg, i.v.) at 22 hours after reperfusion. A–D: Dose-effect relationship of TR (A–B) and ED (C–D) on infarct size and neurological score. Bar graphs showing infarct size (A, C) and neurological score (B,D). E: Dose-effect curves based data from A to D showing effects of TR and ED on infarct size. F–G: Bar graphs showing SOD activity (F) and MDA concentration (G) in the ipsilateral hemisphere. H: Bar graph showing Evans blue contents in the contralateral and ipsilateral hemispheres. ED, edaravone. In A–B, n = 12–13 per group; In C–D, n = 11 per group; In F–G, n = 7 per group; In H, n = 8–11 per group. In A–B, C–D and F–H, ***P < 0.001 vs. Sham; ^#^P < 0.05, ^##^P < 0.01, ^###^P < 0.001 vs. Vehicle; ^$^P < 0.05 vs. 6 mg/kg ED. In E, **P < 0.01. MDA: malondialdehyde; SOD: superoxide dismutases.

Given the data that proved TR and edaravone could scavenge ROS (***Fig. 1***) and the reports that edaravone could prevent cerebral ischemia^[17^,^19]^, we explored whether TR exerts neuroprotective effects against acute ischemic injury. We induced tMCAO in rats, treated the rats with TR or edaravone immediately after reperfusion, and tested the neurological outcome and infarct size at 24 hours after reperfusion. TR significantly produced dose-dependent decreases in infarct size and neurological deficits (***Fig. 2A, B***), so did edaravone (***Fig. 2C, D***), at least in lower doses (0.75‒6 mg/kg). To evaluate the effects of TR and edaravone, we plotted a dose-infarct size inhibition curve (***Fig. 2E***), and found that TR showed better efficacy in inhibiting acute ischemic injury (***Fig. 2E***). SODs, as antioxidant enzymes, play a pivotal role in maintaining redox homeostasis under physiological conditions^[9]^. ROS-induced lipid peroxidation was well established after stroke, and the unstable lipid peroxides decomposed to form MDA^[27]^, thus making MDA a marker of lipid peroxidation. Interestingly, we found that both TR and edaravone substantially reversed the ischemia-induced dysfunctions of SODs and MDA (***Fig. 2F, G***). To test the BBB permeability, we infused the two drugs and Evans blue into tMCAO rats immediately and at 22 hours after reperfusion, respectively, and measured the Evans blue concentration in the contralateral and ipsilateral hemisphere at 24 hours after reperfusion. Consistent with the previous reports^[28]^, ischemia dramatically increased BBB permeability in the ipsilateral hemisphere, and more importantly, TR and edaravone rescued ischemia-induced BBB dysfunction (***Fig. 2H***). Notably, TR showed even better neuroprotection than edaravone (***Fig. 2E‒H***). Together, TR prevents cerebral ischemia *via* inhibiting oxidative stress and maintaining BBB integrity.

### TR has a 2-hour therapeutic window after reperfusion

**Fig.3:**
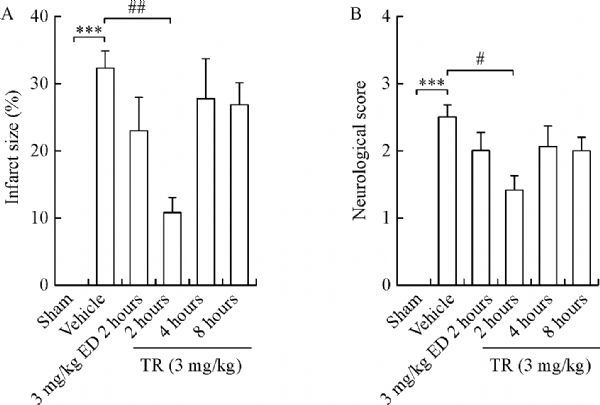
**TR has a therapeutic time window of 2 hours after reperfusion.** tMCAO was induced in rats. TR was given (3 mg/kg, i.v.) at 2, 4 or 8 hours after reperfusion, whereas ED was infused (3 mg/kg, i.v.) at 2 hours after reperfusion, as a positive control. Infarct size and neurological score were measured at 48 hours after reperfusion. Bar graphs showing infarct size (A) and neurological score (B). ED: edaravone. n = 7–11 per group; ***P < 0.001 vs. Sham; ^#^P < 0.05, ^##^P < 0.01 vs. Vehicle.

Previously, we reported that the therapeutic window of edaravone is 2 hours after reperfusion^[19]^. We chose edaravone (2 hours) as a positive control in this study. Unfortunately, edaravone (2 hours) displayed reduction trends, without significant decreases in infarct size or neurological deficits, probably owing to the differences in edaravone doses (3 mg/kg in the present study, 6 mg/kg in the previous study)^[19]^. TR (3 mg/kg, 2 hours) showed robust neuroprotection against ischemic injury, reflected by significant reductions in infarct size and neurological scores (***Fig. 3A, B***). Thus, we concluded that the therapeutic window of TR was 2 hours after reperfusion in our experimental conditions.

### TR significantly attenuates permanent ischemic injury

**Fig.4:**
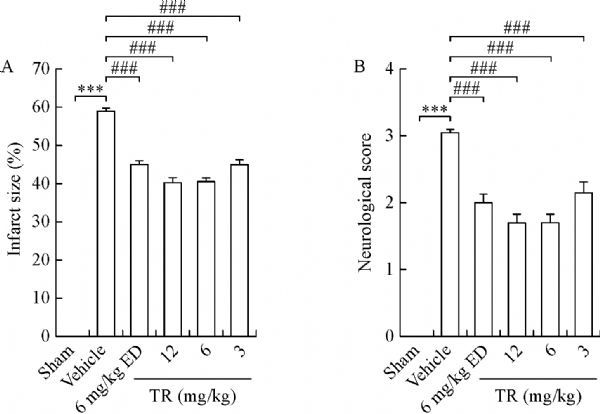
**Effects of TR on permanent ischemia.** Rats were subjected to pMCAO without reperfusion to prepare permanent ischemia models. TR and ED at the indicated doses were given (i.v.) at 2 hours after ischemia beginning. Infarct size and neurological score were measured at 24 hours after pMCAO. Bar graphs showing infarct size (A) and neurological score (B). ED: edaravone; pMCAO: permanent middle artery occlusion; n = 10 per group; ***P < 0.001 vs. Sham; ^###^P < 0.001 vs. Vehicle.

Considering that some stroke patients are not able to receive thrombolytic therapy and thrombectomy, we induced permanent middle artery occlusion (pMCAO) and re-evaluated the efficacy of TR in pMCAO rats. Consistent with the results from the tMCAO model, we found that TR partially reduced permanent ischemia-induced infarct size and neurological deficits in a dose-dependent manner (***Fig. 4***), thereby suggesting that TR prevented permanent ischemia. We also observed that TR (6, 12 mg/kg) slightly decreased infarct size and neurological deficits, compared to edaravone, supporting that the effectiveness of TR in neuprotection was more pronounced.

### TR has long-term protective effects

**Fig.5:**
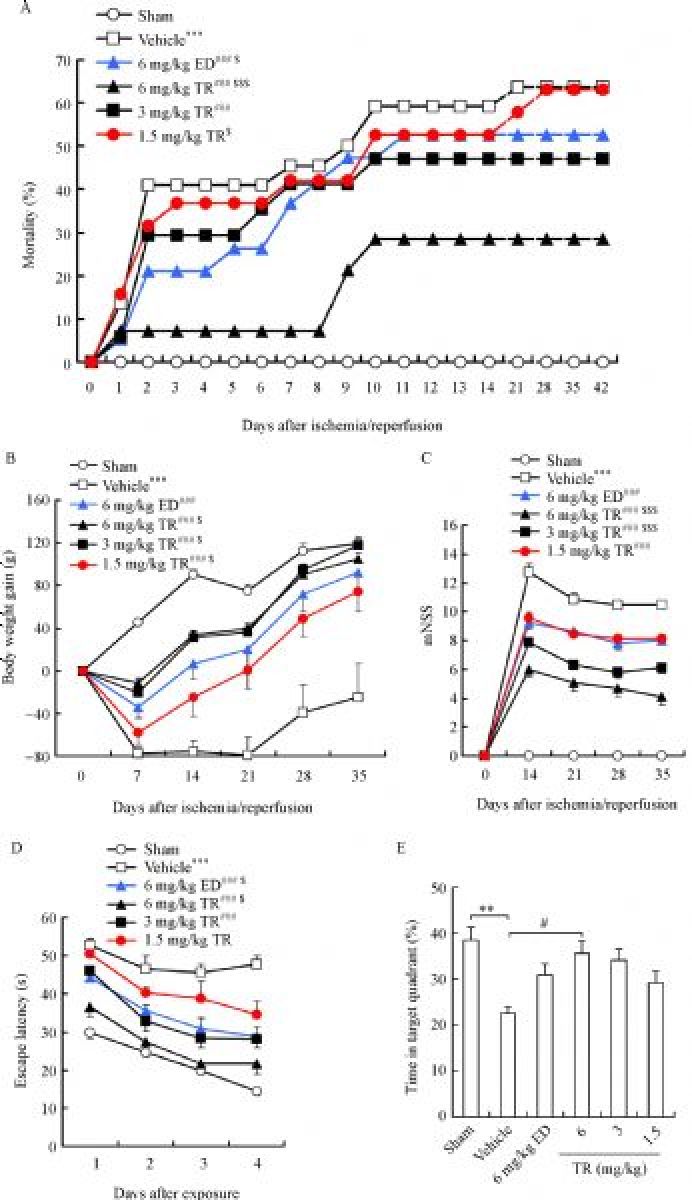
**Long-term protective effects of TR on ischemia-induced impairments in sensorimotor function and spatial memory.** Rats were subjected to tMCAO for 120 minutes, treated with ED or TR (i.v.) at the indicated doses immediately and from 1 to 14 days after reperfusion once daily. Sensorimotor and spatial cognitive functions were detected at the indicated time. A: Time course of the rats’ mortality (at 0 day, *n* = 10 for sham, *n* = 14–22 for other groups; at 42 days, *n* = 10 for sham, *n* = 7–10 for other groups). B: Time course of rats’ body weight gain (n = 7–10 per group). C: Modified neurological severity scores (mNSS) measured at 14, 21, 28, 35 days after reperfusion (n = 7–10 per group). D–E: Escape latency (D) measured from 37 to 40 days and time spent in the target quadrant (E) measured at 41 days after reperfusion in Morris water maze (MWM) task (*n* = 7–10 per group). ED: edaravone. **P < 0.01, ***P < 0.001 vs. Sham; ^#^P < 0.05, ^###^P < 0.001 vs. Vehicle; ^$^P < 0.05, ^$$$^P < 0.001 vs. 6 mg/kg ED.

To investigate the long-term effects of TR, we induced tMCAO in rats, treated the rats with edaravone or TR immediately and from 1 to 14 days after reperfusion once daily, and detected sensorimotor function and spatial memory. Edaravone (6 mg/kg) group was set as a positive control. TR decreased mortality in a dose-dependent manner (***Fig. 5A***). Ischemia significantly resulted in growth retardation and impairments in sensorimotor function and spatial memory (***Fig. 5B‒E***). Notably, TR was shown to markedly reverse the ischemia-induced growth retardation (***Fig. 5B***), neurological deficits (***Fig. 5C***) and prolonged escape latency in the Morris water maze (MWM) task (***Fig. 5D***), dose-dependently, so was edaravone. In MWM task, we also found that TR (6 mg/kg) strongly prolonged the time in the target quadrant (***Fig. 5E***). More importantly, these results indicate that TR (6 mg/kg) further improves sensorimotor function and spatial memory (***Fig. 5C‒E***), compared to Vehicle and edaravone (6 mg/kg). Taken together, these findings suggest that TR could be a more preferable option for stroke treatment, compared to edaravone.

## Discussion

To date, stroke remains a life-threatening disease and leads to high mortality and morbidity worldwide. Currently, intravenous administration of tPA is the mainstay of acute stroke therapy^[2]^. Despite its confirmed efficacy, tPA has been reported to have some toxic effects^[17^,^29]^, such as infarct volume expansion, hemorrhagic transformation and BBB disruption. Neuroprotection is an alternative treatment for AIS. It has been well known that mild hypothermia and drugs targeting post-synaptic density-95 are two neuroprotective strategies for acute ischemic injury^[30]^. However, the successful application of these two strategies are merely in animal models, most clinical trials are uninspiring^[3]^. Therefore, new pharmacological targets need to be developed for AIS treatment.

The mechanisms underlying the stroke-induced neurological deficits are multifaceted. Although *N*-methyl-*D*-aspartate receptor-mediated excitotoxicity has been considered as a major contributor, oxidative stress plays a pivotal role in the pathologenesis of cerebral ischemia^[4]^. Oxidative stress is closely related to some neurodegenerative diseases other than stroke^[31]^, including Alzheimer’s disease and Parkinson’s disease. Under physiological conditions, ROS, including O_**2**_^·−^, HO**·** and H_2_O_2_, are used for maintaining redox homeostasis and other essential biological processes^[9]^. However, excessive oxidative stress may lead to damage to lipids, proteins and DNA^[5]^, and cause pathological changes. Ischemia-induced ROS overproduction leads to further damages to neuronal cells^[32]^, resulting in cerebral cytotoxic edema and infarction. Thus, scavenging ROS can be considered as a strategy for AIS treatment.

In both animal experiments and clinical researches, edaravone has been systematically demonstrated to exert neuroprotective effects against acute ischemic injury by scavenging ROS^[18^‒^19^,^21^‒^22]^. Since 2001, edaravone, as a neuroprotectant, has been given a grade B recommendation in clinical treatment of AIS in Japan^[33]^. ONOO^−^, a stronger oxidative radical leading to protein nitration and dysfunction^[9]^, is mainly generated by the chemical reaction between O_**2**_^·−^ and NO. edaravone has been reported to have potent scavenging activity for ROS, but no effects on O_**2**_^·−[17^,^19]^. Thus its potency is quite limited. Fortunately, in the present study, we found that TR, a novel compound structurally related to edaravone, possesses 2.4-fold higher scavenging ability for O_**2**_^·−^ than edaravone, whereas the eliminating effects of TR and edaravone on HO**·** are comparable, suggesting TR has more potent scavenging effect on ROS. Expectedly, TR showed better efficacy on rescuing neurological deficits and ischemic injury. More importantly, it had further improved behavioral results, compared to edaravone. In this study, we also observed that TR treatment strengthened the antioxidant system, indicative of increased SODs, decreased MDA, and BBB integrity. Thus, TR could be a preferable option for AIS treatment.

Edaravone in combination with tPA is widely used in clinical practice^[18^,^34^‒^35]^, and exerts synergetic neuroprotection on stroke patients. Multiple reasons are responsible for the synergetic effect. Firstly, tPA accelerates recanalization of the occluded artery, thus promoting the access of neuroprotectants in adequate concentrations to the ischemia area^[23]^. Secondly, ROS accounts for reperfusion-induced secondary damage and brain edema^[32]^. Edaravone rescues the damage and brain edema *via* scavenging ROS^[17]^. Thirdly, edaravone decreases the risk of symptomatic intracerebral hemorrhage and BBB permeability^[18]^. Thus, TR combined with tPA may provide a stronger synergetic effect against AIS.
